# Perioperative Myocardial Injury and Acute Kidney Injury in Patients Undergoing Hepatic Resection: Incidence, Risk Factors, and Effects on Outcomes

**DOI:** 10.3390/jcm14176080

**Published:** 2025-08-28

**Authors:** Taner Abdullah, Mert Şentürk, Hürü Ceren Gökduman, İşbara Alp Enişte, İlyas Kudaş, Özgür Bostancı, Erdem Kınacı, İlgin Özden, Funda Gümüş Özcan

**Affiliations:** 1 Department of Anesthesiology and Reanimation, Başakşehir Çam & Sakura City Hospital, 34480 Istanbul, Türkiye; taner.abdullah@gmail.com (T.A.); cerengokduman@hotmail.com (H.C.G.); alpeniste@gmail.com (İ.A.E.); fgumus@hotmail.com (F.G.Ö.); 2 Department of Anesthesiology and Reanimation, School of Medicine, Acıbadem University, 34750 Istanbul, Türkiye; 3 Department of General Surgery, Başakşehir Çam & Sakura City Hospital, 34480 Istanbul, Türkiye; ilyaskudas@hotmail.com; 4 Liver Transplantation & Hepatopancreatobiliary Surgery Unit, Department of General Surgery, Istanbul Başakşehir Çam & Sakura City Hospital, 34480 Istanbul, Türkiye; drbostanci@gmail.com (Ö.B.); erdemkinaci@gmail.com (E.K.); iozden@hotmail.com (İ.Ö.)

**Keywords:** perioperative myocardial injury, perioperative acute kidney injury, hepatic resection

## Abstract

**Background/Objectives:** Perioperative organ injury (POI) is frequently observed following hepatectomy as acute kidney injury (AKI), perioperative myocardial injury (PMI), or both. We aimed to determine the incidences of POI, PMI, and AKI, reveal the risk factors and predictive tools for POI occurrence, and evaluate the relationship between POI and patient outcomes. **Methods:** This was a single-center historical cohort study of consecutive patients. The primary endpoint was the occurrence of POI within 3 days following hepatectomy. **Results:** Out of 128 patients, POI, PMI, and AKI occurred in 48 (37.5%), 36 (28.1%), and 23 (18%) patients, respectively. Ten (7.8%) patients suffered from both PMI and AKI. The presence of chronic kidney disease or systolic/valvular heart disease, fluid balance more than 365 mL/h, and intraoperative bleeding more than 950 mL were the risk factors for POI. A tool created by using the intraoperative decline of central venous oxygen saturation and lactate value during skin closure performed well in predicting POI (area under the ROC curve: 0.79, *p* < 0.001). In patients with POI, the number of those who needed intensive care unit (ICU) follow-up for more than 1 day was significantly higher (21% vs. 6%, *p*: 0.01). The length of hospital stay for these patients was significantly longer as well (11 (8–18) vs. 9 (7–13) days, *p*: 0.02). Two patients (20% of 10 patients who suffered from both AKI and PMI) died in the 90-day follow-up. **Conclusions:** POI is a common complication following hepatectomy and is associated with longer hospital and ICU stays. Patients who suffer from both AKI and PMI have a higher risk of mortality.

## 1. Introduction

Hepatic resections are highly invasive major surgical procedures and are associated with a higher incidence of end-organ injuries, specifically acute kidney injury (AKI) and perioperative myocardial injury (PMI) [1,2].

Perioperative AKI is a well-recognized and widely studied complication in patients undergoing hepatic resection [3,4,5]. The incidence of AKI in this patient group varies between 9% and 28% depending on the surgical clinics, characteristics of included patients, and the extent of the surgeries included in the analyses [3,4,5]. Development of AKI has been shown to be associated with higher morbidity, longer hospital stays, and higher in-hospital and long-term mortality rates [5,6].

Unlike AKI, PMI is scarcely studied in this patient group, as it has been defined quite recently [7,8]. The pooled incidence of PMI in patients undergoing surgery is 18% [9]. Regarding hepatic resection, there is only one small prospective study performed with 18 patients, and in that study, the incidence has been reported as 28% [2]. Considering that PMI has been associated with increased morbidity and mortality [8,9] (even if the criteria for acute coronary syndrome are not met), there is a need for studies to better understand the importance of PMI in patients undergoing hepatectomy.

Impaired oxygen demand-supply ratio during the perioperative period is the main reason for the development of both AKI and PMI [10,11]. Due to their common etiology and relationship with worsened outcomes, we propose to pool these two endpoints under the definition of perioperative organ injury (POI).

The primary aims of this study were to determine the incidence of AKI, PMI, and POI, and reveal the risk factors as well as predictive tools for the development of POI in patients undergoing hepatic resection. Secondary aims were to evaluate the relationship between the occurrence of POI and length of hospital and intensive care unit (ICU) stay and 90-day mortality.

## 2. Materials and Methods

### 2.1. Study Design and Patient Inclusion

This study was designed as a single-center historical cohort study of consecutive patients undergoing hepatic resection between June 2021 and June 2024 at the Hepatopancreaticobiliary Surgery (HPB) Clinic of Basaksehir Çam and Sakura City Hospital, Istanbul, Turkiye. We identified the patients from a prospectively maintained data file that includes the perioperative data of patients undergoing surgery at this center. Ethical approval for the study design and data analysis was obtained from the Clinical Research Ethics Committee of Başaksehir Çam and Sakura City Hospital (number: 2024.297, date: 16 December 2024), and the written informed consent was waived. All methods were carried out in accordance with the Declaration of Helsinki. The study protocol was registered at https://clinicaltrials.gov (NCT06775158) before the screening began. All patients above the age of 18 were included, and those with the following conditions were excluded: presence of end-stage kidney disease, emergency surgery, application of total vascular exclusion, concomitant gastrointestinal resection, and non-neoplastic procedures.

### 2.2. Perioperative Management

We have a standardized management protocol for hepatic resection surgeries, and all cases are managed by the same three anesthesiologists (TA, HCG, and İAE). Following the monitoring of peripheral oxygen saturation, electrocardiogram, blood pressure, and patient state index (PSI) (Masimo Inc., Irvine, CA, USA), the left radial artery is catheterized using a 20-gauge arterial catheter (Vygon, Padova, Italy) under mild sedation. The square-wave test is performed to confirm the absence of overdamping and underdamping in the arterial pressure waveform. Thereafter, the arterial line is connected to an arterial waveform analysis device (MostCare monitor, Vygon, Padova, Italy) for the continuous measurement of cardiac output and dynamic parameters.

Anesthesia induction is performed with 1% propofol accompanied by 1 mcg/kg fentanyl and 0.6 mg/kg rocuronium bromide. The dose of propofol is titrated by aiming at a PSI value below 50. Sevoflurane (1–2%) and remifentanil (0.03–0.3 mcg/kg/min) infusions are used for the maintenance of anesthesia, targeting a PSI of 25–50. Neuromuscular blockade is maintained with 0.1 mg/kg rocuronium bromide boluses administered every 30 min. Pharyngeal temperature is monitored in all patients and maintained at 36–37 °C.

Mechanical ventilation is administered using volume-controlled ventilation (Perseus A500, Drager, Lübeck, Germany) with a tidal volume of 8 mL/kg of the ideal body weight at a rate of 12–15 breaths per minute and a mixture of 40% oxygen and air, with a positive end-expiratory pressure of 4–6 cmH_2_O.

The right internal jugular vein is catheterized using an 8.5 French central venous catheter for monitoring central venous pressure (CVP), sampling central venous blood, and administering treatments. Arterial and venous blood samples are collected simultaneously every 120 min or more frequently if required for hemodynamic management.

A balanced crystalloid solution is used for both maintenance and bolus fluid replacement. Colloids are only used when there is hypotension due to ongoing bleeding, and the usage is limited to 20 mL/kg. The hemoglobin threshold for packed red blood cell transfusion varies between 7 and 9 g/dL in accordance with patients’ comorbidities, hemodynamic and laboratory parameters, and whether there is ongoing bleeding. Mean arterial pressure is strictly maintained above baseline MAP × 0.8 and ≥65 mmHg in every patient. Baseline MAP is identified in line with the blood pressure recordings on the day before the surgery on the surgical ward. We do not perform a low CVP protocol per se. Instead, we apply goal-directed protocols by using the parameters obtained from arterial waveform analysis to avoid unnecessary fluid resuscitation and assure sufficient perfusion. Briefly, in the presence of a low cardiac index (<2.2 L/min/m^2^) or at least two signs of hypoperfusion—such as pulse pressure variation ≥ 10%, oliguria (<0.3 mL/kg/h), hemoconcentration (increase in hematocrit without blood transfusion), lactate increase >1 mmol/L with a minimum absolute value of 2 mmol/L, a negative trend in base excess with negative absolute values, or a heart rate increase > 10% while anesthesia depth and catecholamine infusion remain stable—we perform a mini fluid challenge by infusing 100 mL of crystalloids over 1 min to assess fluid responsiveness. If the patient is fluid responsive (defined as an increase in stroke volume index of at least 5% following the mini fluid challenge), 250–500 mL of crystalloids is infused to correct hypoperfusion. If the patient is not fluid responsive, dobutamine infusion is initiated or increased. When low MAP persists despite an adequate cardiac index, noradrenaline infusion is initiated or increased.

All patients are extubated in the operating room and are transferred to the post-surgery ICU dedicated to the HPB clinic, where they spend the first 24 h post-surgery under the surveillance of the same intensivist unless there is a need for a longer duration.

Blood samples for laboratory follow-up (including highly sensitive troponin T [hsTnT] and creatinine) are collected once within the week before the surgery, at admission to the ICU, and on the first three mornings following the surgery day. After the third day, the intervals between the blood samplings are gradually increased unless there is a significant finding.

### 2.3. Surgical Procedure

Surgeries are performed by senior hepatobiliary surgeons (İÖ, EK, and ÖB) by using a right subcostal incision with an upward midline extension. In hemihepatectomy or extended hepatectomy, hilar dissection is performed to ligate the hepatic artery and portal vein branch supplying the lobe of liver to be removed before parenchymal transection. For wedge resection or segmentectomy, intraparenchymal isolation and ligation of the vascular pedicle are performed. Pringle’s maneuver is applied in all procedures if needed. It is achieved by encircling the hepatoduodenal ligament and intermittently applying a vascular clamp (15 min clamping and 5 min unclamping) until the end of transection. Liver transection is performed with an ultrasonic aspirator (Cavitron Ultrasonic Surgical Aspirator, CUSATM; ValleyLab, Boulder, CO, USA). Hemostasis is achieved by cautery, titanium clips, and ligatures. Major hepatic veins are either clamped, divided, and closed with prolene sutures or divided with endovascular staplers (Tyco Healthcare, Norwalk, CT, USA).

### 2.4. Study Endpoints, Definitions, and Calculations

The primary endpoint was the occurrence of POI, defined as the occurrence of AKI and/or PMI. AKI was defined as at least 0.3 mg/dL or a 50% increase from baseline creatinine levels within the first three days after the surgery and staged in line with the KDIGO criteria [12]. PMI was defined as at least a 14 ng/L increase from baseline hsTnT levels within the same time duration. This is the definition stated in the most recent guidelines regarding non-cardiac surgery [7]. The length of ICU and hospital stay and 90-day mortality were determined for the postoperative period. Major hepatic resection was defined as the resection of at least three Couinaud segments [4,13]. Chronic kidney disease (CKD) and chronic hsTnT elevation were defined as a baseline glomerular filtration rate < 60 mL/min/1.73 m^2^ and hsTnT > 14 ng/L, respectively. Systolic/valvular heart disease was defined as an ejection fraction < 50% or the presence of moderate/severe valvular disease during echocardiography. Coronary artery disease was defined as the presence of at least one of the following conditions: a history of angina or acute coronary syndrome, a finding of significant stenosis (≥50% of the diameter of any coronary artery) on coronary angiogram or a segmental cardiac wall motion abnormality on echocardiography, an ECG with pathological Q waves in two contiguous leads, a segmental fixed defect on radionuclide imaging, or any positive cardiovascular stress test demonstrating cardiac ischemia. Blood loss was calculated by using the intraoperative hematocrit change in line with the formula defined by Lopez-Picado et al. [14].

### 2.5. Statistical Analysis

The distribution of interval data was assessed using the d’Agostino-Pearson test. Data that followed a normal distribution are presented as mean ± standard deviation, while non-normally distributed data are shown as median (25th percentile–75th percentile). Student’s *t*-test and the Mann–Whitney u test were used for the comparison of the variables in line with their distributions. Categorical data are expressed as a number (percentage) and were compared with the chi-square test. Logistic regression analysis was performed by using statistically significant patient- and surgery-dependent variables for determining the risk factors for POI. Another model was created to evaluate the ability of intraoperative laboratory changes in predicting the occurrence of POI by using receiver operating characteristic (ROC) curves. Only statistically significant variables were used. To perform logistic regression analyses, statistically significant continuous variables were converted into categorical variables by using the Youden index (sensitivity + specificity − 1) and upper and lower points of the gray zones (points where positive likelihood ratio is 0.1 and negative likelihood ratio is 10). These thresholds were determined by applying the ROC curve analysis for each variable [15]. When similar statistically significant variables were present (i.e., the last, the highest, and delta values of lactate), the one with the highest value of area under the ROC curve (ROCAUC) was used in the logistic regression. The 90-day mortality was analyzed using the Kaplan–Meier survival analysis and compared using the log-rank test.

The sufficiency of the sample size was evaluated post hoc. According to the Green formula for the sample size of a logistic regression model, the sample size was sufficient to evaluate up to nine variables in a model (N ≥ 50 + 8* number of variables) [16]. For the given sample size and incidence, the margin of error for the incidence of POI was calculated as 8.4%.

Statistical analyses were conducted using SPSS for Windows version 21.0 (SPSS Inc., Chicago, IL, USA) and MedCalc version 16.1 (MedCalc Software Ltd., Ostend, Belgium).

## 3. Results

**Figure 1:**
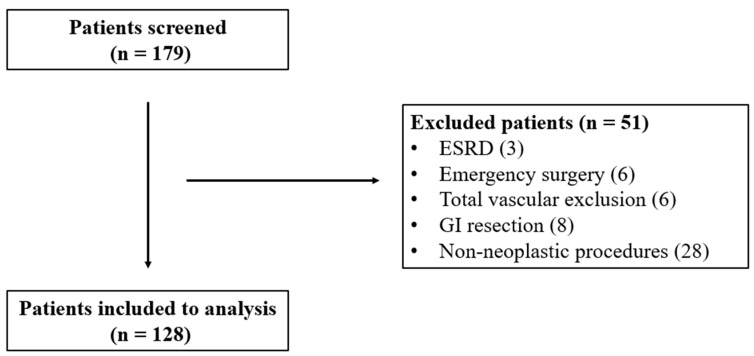
Study flow-chart. ESRD: end-stage renal disease.

A total of 179 patients met the inclusion criteria. Of them, 128 patients were included in the analysis (Figure 1). POI was observed in 48 (37.5%) patients (29.1–45.9% with an 8.4% margin of error). PMI and AKI occurred in 36 (28.1%) and 23 (18%) patients, respectively. Of them, 10 (7.8%) patients suffered from both PMI and AKI. Among those who suffered from AKI, 13 (72%) patients were diagnosed as stage 1, and 5 (28%) as stage 2. Based on the development of POI, patients were allocated into two groups (POI+ and POI−) and compared.

Data regarding patients’ demographic characteristics, comorbidities, medications, and surgical diagnoses are given in Table 1, while the parameters regarding the surgery and perioperative management are presented in Table 2. The results of the logistic regression analysis performed with the variables that are significant in Table 1 and Table 2 are presented in Table 3. The presence of CKD and systolic/valvular heart disease, fluid balance more than 365 mL/h, and intraoperative bleeding more than 950 mL were determined as the risk factors for the development of POI. The ROCAUC of the logistic regression model created with these variables was 0.87 (95% CI: 0.80–0.92, *p* < 0.001, Table 3). A total of 80% of the cases were classified correctly with this model.

**Figure 2:**
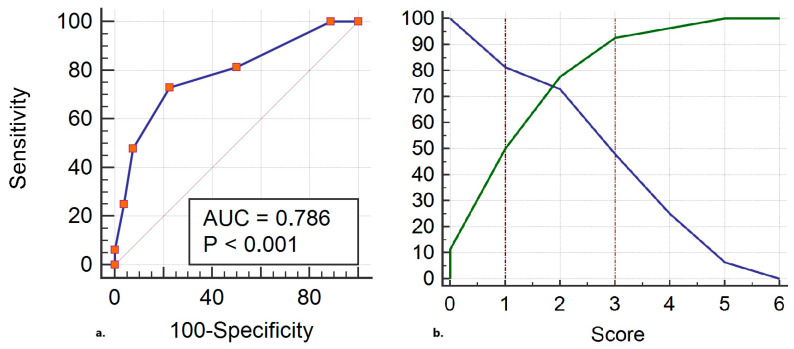
(**a**) Receiver operating characteristic curve generated by using the difference between the first and worst values of central venous oxygen saturation and lactate value at the end of the surgery. The best cut-off value: >2, sensitivity: 73%, specificity: 78%. (**b**) Gray zone analysis for the same model. The length of ICU stay of the cohort was 1 (1–1) day. A total of 113 (88%) patients needed only 1-day ICU follow-up. The number of patients who needed ICU surveillance longer than 1 day was significantly higher in the POI+ group than that in the POI− group [10 (21%) vs. 5 (6%) patients, respectively; *p*: 0.01].

Intraoperative laboratory values of the groups are presented in Table 4. All lactate variables and the difference between the first and worst values of central venous oxygen saturation (ScvO_2pre-lowest_) were statistically different between the groups. A model was constructed by using ScvO_2pre-lowest_ (points: 0: ScvO_2_ = 0, 1: 0 < ScvO_2_ ≤ 3.4, 2: 3.4 < ScvO_2_ ≤ 4, 3: ScvO_2_ > 4) and the lactate value at the end of the surgery (Lactate_post_, points: 0: lac ≤ 1, 1: 1 < lac ≤ 3.1, 2: 3.1 < lac ≤ 4.9, 3: lac > 4.9) and evaluated in terms of predicting POI. The ROCAUC of this model was 0.79 (95% CI: 0.71–0.85, *p* < 0.001, Figure 2a). A total of 76% of the cases were classified correctly with this model. Gray zone analysis revealed that 26 (20%) patients who had scores below 3 and above 1 were inside the gray zone area (Figure 2b).

The length of hospital stay of the cohort was 9 (7–14) days. POI+ patients needed significantly longer duration of hospital stay than POI− patients [11 (8–18) vs. 9 (7–13) days for POI+ and POI− groups, respectively; *p*: 0.02].

**Figure 3:**
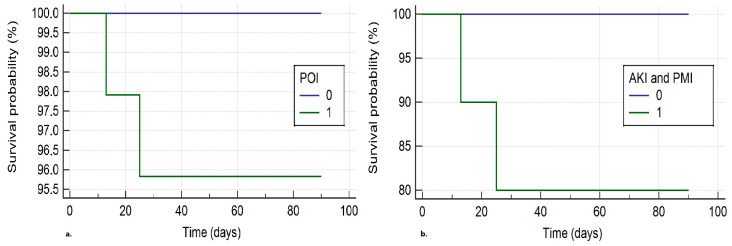
(**a**) Kaplan–Meier plots for survival probability up to 90 days after surgery of patients who did and did not suffer from perioperative organ injury (POI). (**b**) Kaplan–Meier plots for survival probability up to 90 days after surgery of patients who suffered from both acute kidney injury (AKI) and perioperative myocardial injury (PMI), and those who suffered from none or one organ injury. 0: Absence of POI (**a**) or AKI and PMI (**b**). 1: Presence of POI (**a**) or AKI and PMI (**b**).

Two patients died in the 90-day follow-up. One died due to cardiac-related causes on day 25, and the other due to postoperative liver failure (PLF) on day 13. The second patient was the only one in the cohort who suffered from PLF within three days following surgery (defined as an international normalized ratio > 2). These patients were 2 (20%) of the 10 patients who suffered from both AKI and PMI within the first three days postoperatively. There was no mortality observed among the patients who suffered from no or one organ injury. The survival analyses for POI+ and POI− groups were not statistically different (log-rank test: chi-squared statistic: 3.4, *p*: 0.07, Figure 3a). However, when patients who suffered from both AKI and PMI were compared with the rest of the cohort, the survival curves were significantly different (log-rank test: chi-squared statistic: 24.9, *p* < 0.001, Figure 3b).

## 4. Discussion

POI occurred in 48 (37.5%) patients. Of them, 23 (18%) patients suffered from AKI, while PMI was observed in 36 (28.1%) patients. Ten (7.8%) patients presented with both AKI and PMI. Risk factors for the development of POI were the presence of CKD and systolic/vascular heart disease, blood loss of more than 950 mL, and positive fluid balance of more than 365 mL per hour. POI development was associated with longer ICU and hospital stays. Two patients died during the follow-up, and both were among the 10 patients (20%) who suffered from both PMI and AKI.

The incidence of AKI reported in this study is higher than the incidence reported for major abdominal surgeries [1]. However, it is comparable with most of the studies that were focused on high-risk patients or those undergoing hepatic resection [4,6,17]. Similarly, the incidence of PMI is higher than the reported pooled incidence [9]. There is only one study that has previously focused on the incidence of PMI in patients undergoing hepatectomy [2]. Although the sample size of that study was very limited, the incidence they reported is comparable (5 [28%] out of 18 patients) with that of the current study. To our knowledge, this is the first study with a large sample size that reports a PMI incidence for hepatic resections.

Considering that POI occurs due to a deranged oxygen supply–demand ratio [10,11], it is not surprising to find higher frequencies of POI in patients undergoing hepatic resection, as these surgeries involve increased risk for bleeding and substantial fluid shifts [1]. Indeed, both blood loss of more than 950 mL and positive fluid balance of more than 365 mL/h were found as risk factors for POI in the current study. Previous studies have demonstrated a U-shaped relationship between perioperative complications and fluid balance [18]. Irrespective of the reason, positive fluid balance has the potential to result in tissue edema, which has a negative effect on the perfusion pressure of the tissue, causing a reduction in oxygen supply.

In the current study, chronic kidney disease and systolic/valvular heart disease were the comorbidities determined as independent risk factors for POI. As previous studies demonstrated and the current study confirmed, baseline kidney function has a strong association with both AKI and PMI [6,7]. On the other hand, structural heart diseases can diminish the oxygen supply by reducing the stroke volume and can result in increased myocardial oxygen demand due to altered filling pressures and volumes [19].

Previous studies demonstrate contradictory results regarding the association between several perioperative features (duration of surgery, application of major resection, application and duration of Pringle maneuver) and POI, specifically AKI [3,5,17,20]. Although we were unable to reveal such an association in our study, the accuracy of the current study’s model (ROCAUC: 0.88) was higher than all other studies. Considering the contradictory results and the accuracy of the current model, we think it is reasonable to claim that, as long as the anesthetic and surgical techniques are performed with the aim to prevent significant bleeding and positive fluid balance, the duration or majority of the surgery and the duration of the Pringle maneuver might not have a significant impact on the development of POI.

Two patients died within the 90-day follow-up in this cohort. They were 2 of the 10 patients who suffered from both AKI and PMI (20% of patients with both organ injuries). To our knowledge, this is the first study that demonstrates such a strong relationship between mortality and POI. Although AKI and PMI are separately associated with increased mortality [6,8], there is no study that evaluates these endpoints together or concomitantly. The mortality rate of 20% in patients diagnosed with both AKI and PMI in the current study supports that patients who suffer from AKI or PMI and die during the follow-up might be the ones who suffer from other organ injury as well. Therefore, we strongly believe that future studies should focus on the concomitant occurrence of AKI and PMI.

Lactate and ScvO_2_ are known as good indicators of the oxygen supply/demand ratio [21,22]. Consequently, previous studies regularly demonstrated that perioperative lactate values are associated with postoperative morbidity and mortality [22,23,24,25]. Unlike lactate, data regarding ScvO_2_ is limited. There is only one study that investigated the relation between ScvO_2_ and outcomes in hepatic resection patients [21]. In that study, the authors used a 10% decline in ScvO_2_ as the threshold, considering previous reports, and demonstrated that a decline in ScvO_2_ of more than 10% is associated with increased morbidity. In line with the literature, the current study revealed that a model constructed by using lactate_post_ and ScvO_2pre-lowest_ might be used for predicting the occurrence of POI. Nevertheless, our results demonstrate that the risk increases with much lower ScvO_2_ declines (ScvO_2pre-lowest_) values in comparison to the previous report [21].

The main strength of this study is the significant homogeneity of the cohort regarding its perioperative management, as the time window is relatively short. Moreover, a systematic patient follow-up registry prevented significant data losses. Additionally, all patients were managed by the same surgery and anesthesia teams that are in substantial agreement regarding the perioperative management protocol. Nevertheless, while this enhances the uniformity of perioperative management, it may limit the external validity of the study. Also, since the prospectively maintained data file was designed in accordance with the anesthesiologist’s perspective and therefore mainly focused on the intraoperative and early postoperative period, it did not include patient follow-up data beyond the third day of the surgery. This hindered our ability to report high-quality data on surgery-related complications. There are other limitations that need to be addressed as well. First, this was a single-center retrospective cohort study with a relatively small sample size regarding a specific surgical procedure. Therefore, the results should be extrapolated cautiously. Validation of the suggested predictive tools requires prospective multi-center studies with larger sample sizes. Second, we were unable to collect intraoperative hemodynamic data and therefore could not evaluate the relation between hemodynamic instability and bleeding/positive fluid balance. Third, we did not collect the data regarding the fluid management in the ICU and the surgical ward. Fourth, the definition of PMI varies widely in the literature. Although we adopted the most recent guidelines, applying different definitions might affect the results. Fifth, we utilized ScvO_2_ to develop a predictive tool. Yet, the application of a central venous line may not be a part of standard care in all centers. Lastly, data regarding the blood loss was estimated (not measured) by using the formula defined by Lopez-Picado.

## 5. Conclusions

POI is a common complication following hepatectomy. Risk factors are the presence of CKD and systolic/valvular heart disease, as well as perioperative increased blood loss and fluid balance. POI is associated with longer hospital and ICU stays. Patients who suffer from both AKI and PMI have a higher risk of mortality. Lactate_post_ and ScvO_2pre-lowest_ can be used for predicting the POI occurrence. Future studies should focus on the concomitant occurrence of AKI and PMI.

## Figures and Tables

**Table 1:** Characteristics of patients.

	All (128)	Injury + (48)	Injury − (80)	*p* Value
**Sex**				0.46
Male	75 (59%)	26 (54%)	49 (61%)	
Female	53 (41%)	22 (46%)	31 (39%)	
**Age (years)**	57 ± 13	59 ± 13	56 ± 13	0.30
**Weight (kg)**	75 ±12	76 ± 12	75 ± 12	0.60
**Height (cm)**	167 ± 9	166 ± 9	167 ± 9	0.70
**Comorbidities**				
Hypertension	58 (45%)	27 (56%)	31 (39%)	0.06
Diabetes mellitus	30 (23%)	13 (27%)	17 (21%)	0.45
Coronary artery disease	20 (16%)	6 (13%)	14 (18%)	0.45
Systolic/Valvular Heart Disease	7 (6%)	6 (13%)	1 (1%)	**0.007**
Systolic heart disease	2 (1.6%)	2 (4%)	0 (0%)	0.07
Valvular heart disease	5 (4%)	4 (8%)	1 (1%)	0.04
Cerebrovascular disease	2 (2%)	1 (2%)	1 (1%)	0.71
Pulmonary disease	2 (2%)	1 (2%)	1 (1%)	0.71
Thyroid disease	17 (113%)	6 (13%)	11 (14%)	0.84
Chronic Kidney Disease	9 (7%)	7 (15%)	2 (3%)	**0.01**
Chronic hsTnT Elevation	19 (15%)	10 (21%)	9 (11%)	0.14
**ASA Score**				0.48
1	25 (20%)	6 (12%)	19 (24%)	
2	81 (63%)	33 (69%)	48 (60%)	
3	19 (15%)	8 (17%)	11 (14%)	
4	3 (2%)	1 (2%)	2 (2%)	
**Medications**				
Beta blocker	27 (21%)	13 (27%)	14 (18%)	0.20
ACEi/ARB	33 (26%)	16 (33%)	17 (21%)	0.13
CCB	32 (25%)	15 (31%)	17 (21%)	0.21
Statin	12 (9%)	7 (15%)	5 (6%)	0.12
Antiplatelet Therapy	20 (16%)	7 (15%)	13 (16%)	0.80
NOACs	1 (1%)	1 (2%)	0 (0%)	0.20
OAD	30 (23%)	13 (27%)	17 (21%)	0.45
Insulin	5 (4%)	1 (2%)	4 (5%)	0.41
**Surgical Diagnosis**				0.19
Hepatocellular carcinoma	55 (43%)	19 (40%)	36 (45%)	
Hepatic metastasis	37 (29%)	16 (33%)	21 (26%)	
Colangiocellular carcinoma	26 (20%)	12 (25%)	14 (18%)	
Gallbladder malignancy	10 (8%)	1 (2%)	9 (11%)	

Values are expressed as frequency (percentage) and mean ± SD. hsTnT: highly sensitive troponin T, ACEi: angiotensin converting enzyme inhibitors, ARB: angiotensin receptor blockers, CCB: calcium channel blockers, NOAC: new oral anticoagulants, OAD: oral antidiabetic drugs. Statistical significance: ***p* < 0.05**.

**Table 2:** Intraoperative characteristics.

	All (128)	Injury + (48)	Injury − (80)	*p* Value
**Number of segments resected**				**0.01**
1–2 segments	41 (32%)	9 (19%)	32 (40%)	
3–5 segments	87 (68%)	39 (81%)	48 (60%)	
**Duration of anesthesia (min)**	398 (300–470)	403 (350–480)	390 (300–458)	0.13
**Duration of surgery (min)**	360 (270–420)	373 (303–433)	345 (245–420)	0.08
**Use of the Pringle maneuver (min)**	26 (0–60)	38 (0–64)	17 (0–64)	**0.04**
**Anesthesia management**				
×MAC of Sevoflurane (%)	0.70 ± 0.13	0.72–0.15	0.74 ± 0.11	0.26
Total remifentanil dose (mcg/kg/min)	1280 (1000–2000)	1490 (1000–2070)	1200 (920–1800)	0.15
Mean remifentanil dose (mcg/kg/min)	0.05 (0.04–0.06)	0.05 (0.04–0.06)	0.05 (0.04–0.07)	0.56
**Fluids**				
Crystalloids during surgery (mL)	3500 (3000–5000)	4500 (3500–6000)	3000 (2500–4500)	**<0.001**
Total fluids (mL)	3975 (3000–5500)	5050 (3500–6600)	3500 (2500–4500)	**<0.001**
Urine output (mL)	650 (434–1000)	650 (435–1038)	650 (473–1000)	0.82
Estimated blood loss (mL)	288 (100–760)	750 (1000–1311)	208 (100–593)	**0.003**
Fluid Balance (mL)	2453 (1928–3802)	3155 (2385–4481)	2269 (1544–3223)	**<0.001**
Fluid balance per hour (mL)	406 (297–495)	478 (384–581)	353 (278–472)	**<0.001**
Patients received colloids (n)	34 (27%)	18 (38%)	16 (20%)	**0.03**
Patients received PRBC (n)	29 (23%)	18 (38%)	11 (14%)	**0.003**
**Vasopressors and Inotropes**				
Noradrenaline (n)	82 (64%)	37 (77%)	45 (56%)	**0.02**
Noradrenaline ^#^ (mcg/kg/min)	0.14 ± 0.08	0.18 ± 0.08	0.10 ± 0.06	0.07
Dobutamine (n)	24 (19%)	9 (19%)	15 (19%)	1.00
Dobutamine ^#^ (mcg/kg/min)	2.9 ± 1.3	3.4 ± 1.6	2.4 ± 0.7	0.22

Values are expressed as frequency (percentage), mean ± SD, and median (25th to 75th percentile). PRBC: packed red blood cells. ^#^ patients who did not receive the drug were not included in the calculation. Statistical significance: ***p* < 0.05**.

**Table 3:** Multivariable logistic regression analysis for the association between perioperative organ injury and patient characteristics.

	Odds Ratio	95% CI	*p* Value
**Fluid Balance (FB)**			
FB ≤ 300 (mL/h)	1	reference	
300 < FB ≤ 365 (mL/h)	5.5	0.6–52	0.07
365 < FB ≤ 550 (mL/h)	13	2–84.7	**0.007**
FB > 550 (mL/h)	20.7	2.3–185	**0.007**
**Estimated Blood Loss (BL)**			
BL < 750 (mL)	1	reference	
750 ≤ BL ≤ 950 (mL)	3	0.4–24	0.30
BL > 950 (mL)	7.8	2.2–26.9	**0.001**
**pRBC Transfusion (n)**			
0–1	1	reference	
≥2	0.5	0.1–2.6	0.43
**Noradrenaline infusion**	1.5	0.5–4.8	0.49
**Duration of Pringle maneuver**			
0–45 min	1	reference	
>45 min	2.1	0.8–6.1	0.15
**Major Hepatectomy**	1.4	0.4–4.7	0.59
**Chronic Kidney Disease**	28.8	1.7–486.4	**0.02**
**Systolic/Valvular heart disease**	14.7	1.4–159.3	**0.03**

CI: confidence interval, pRBC: packed red blood cells. Statistical significance: ***p* < 0.05**.

**Table 4:** Intraoperative laboratory parameters.

	All (91)	Injury + (48)	Injury − (80)	*p* Value
Hemoglobin_pre_ (g/dL)	11.7 ± 1.7	11.5 ± 2	11.9 ± 1.5	0.21
Hemoglobin_post_ (g/dL)	11.4 ± 1.5	10.8 ± 1.4	11.8 ± 1.5	**<0.001**
Hemoglobin_lowest_ (g/dL)	10.8 ± 1.6	10.1 ± 1.5	11.2 ± 1.6	**<0.001**
Hemoglobin_pre-post_ (g/dL)	0.1 (−0.6–1)	0.3 (−0.35–1.4)	−0.1 (−0.75–0.75)	**0.02**
Hemoglobin_pre-lowest_ (g/dL)	0.5 (0–1.6)	1.1 (0.1–1.9)	0.3 (0–1.1)	**0.01**
Lactate_pre_ (mmol/L)	0.9 (0.6–1.2)	1 (0.7–1.2)	0.8 (0.6–1.3)	0.47
Lactate_post_ (mmol/L)	2.8 (1.7–4.2)	3.4 (2.6–5)	2.5 (1.4–3.4)	**<0.001**
Lactate_highest_ (mmol/L)	3.3 (2–4.9)	3.9 (2.7–5.2)	3 (1.8–4.5)	**0.01**
Lactate_post-pre_ (mmol/L)	1.9 (0.7–3.2)	2.5 (1.4–4.1)	1.5 (0.4–2.5)	**<0.001**
Lactate_highest-pre_ (mmol/L)	2.2 (1–3.8)	3 (1.5–4.3)	2.1 (0.7–3.4)	**0.01**
S_cv_O_2pre_ (%)	78 ± 7	78 ± 6	77 ± 7	0.47
S_cv_O_2post_ (%)	80 ± 6	81 ± 6	80 ± 6	0.45
S_cv_O_2lowest_ (%)	75 ± 7	75 ± 6	76 ± 7	0.23
S_cv_O_2pre-lowest_ (%)	0 (0–3.3)	2.2 (0–6)	0 (0–2)	**<0.001**
S_cv_O_2post-pre_ (%)	2 (−0.7–6.2)	0.9 (−2.5–5.1)	2.1 (0.2–6.5)	0.20
Δ_v-a_Pco_2pre_ (mmHg)	6 (5–7.3)	5.8 (5–6.9)	6.5 (5.3–7.7)	0.10
Δ_v-a_Pco_2post_ (mmHg)	5.5 (4.6–6.5)	5.5 (4.7–6.5)	5.5 (4.5–6.8)	0.67
Δ_v-a_Pco_2highest_ (mmHg)	6.9 (5.9–9)	6.8 (5.7–9)	7 (6–8.8)	0.80
Δ_v-a_Pco_2highest-pre_ (mmHg)	0.3 (0–1.8)	0.5 (0–2.2)	0.2 (0–1.5)	0.18
Δ_v-a_Pco_2post-pre_ (mmHg)	−0.5 (−1.9–0.7)	−0.3 (−2–1.5)	−0.5 (−1.9–0.3)	0.40
Base excess_pre_ (mmol/L)	1.3 (−0.2–2.7)	0.8 (−0.4–2.5)	1.3 (−0.2–2.9)	0.78
Base excess_post_ (mmol/L)	1.2 (−0.2–2.8)	0.5 (−0.5–2)	1.3 (0–3.3)	0.06
Base excess_lowest_ (mmol/L)	−0.9 (−3–0.8)	−1.6 (−3.3–0)	−0.5 (−3–1.1)	0.08
Base excess_pre-lowest_ (mmol/L)	1.6 (0.2–3.4)	2.1 (0.8–3.7)	1.3 (0–3.2)	0.07
Base excess_pre-post_ (mmol/L)	−0.2 (−1.3–1.7)	0.5 (−1.2–2.2)	−0.6 (−1.5–1.2)	0.07

Values are expressed as mean ± SD and median (25th to 75th percentile). _pre_: pre-incision value, _post_: value at the end of the surgery prior to extubation, _lowest_: the lowest value seen in the operating room. _highest_: the highest value seen in the operating room. S_cv_O_2:_ central venous oxygen saturation, Δ_v-a_Pco_2_: arterio-venous difference of carbon dioxide pressure. Statistical significance: ***p* < 0.05**.

## Data Availability

The original contributions presented in this study are included in the Supplementary Materials. Further inquiries can be directed to the corresponding author(s).

## Supplementary Materials

The following supporting information can be downloaded at: https://www.mdpi.com/article/10.3390/jcm14176080/s1.

## Abbreviations

The following abbreviations are used in this manuscript:

AKI	Acute Kidney Injury
PMI	Perioperative Myocardial Injury
POI	Perioperative Organ Injury
ROC	Receiver Operating Characteristics
ICU	Intensive Care Unit
HPB	Hepatobiliary and Pancreas
PSI	Patient State Index
CVP	Central Venous Pressure
MAP	Mean Arterial Pressure
CKD	Chronic Kidney Disease
PLF	Postoperative Liver Failure
